# Predicting Neuropathy and Reactions in Leprosy at Diagnosis and Before Incident Events—Results from the INFIR Cohort Study

**DOI:** 10.1371/journal.pntd.0000500

**Published:** 2009-08-11

**Authors:** W. Cairns S. Smith, Peter G. Nicholls, Loretta Das, Pramila Barkataki, Sujai Suneetha, Lavanya Suneetha, Rupendra Jadhav, P. S. S. Sundar Rao, Einar P. Wilder-Smith, Diana N. J. Lockwood, Wim H. van Brakel

**Affiliations:** 1 University of Aberdeen, Aberdeen, United Kingdom; 2 University of Southampton, Southampton, United Kingdom; 3 Naini Community Hospital, Uttar Pradesh, India; 4 Faizabad Leprosy Hospital, Uttar Pradesh, India; 5 Blue Peter Research Centre, Hyderabad, India; 6 Stanley Browne Laboratory, Miraj, India; 7 The Leprosy Mission, New Delhi, India; 8 National University Hospital, Singapore; 9 London School of Hygiene & Tropical Medicine, London, United Kingdom; 10 Royal Tropical Institute, Amsterdam, The Netherlands; Erasmus MC, Netherlands

## Abstract

**Background:**

Leprosy is a disease of skin and peripheral nerves. The process of nerve injury occurs gradually through the course of the disease as well as acutely in association with reactions. The INFIR (ILEP Nerve Function Impairment and Reactions) Cohort was established to identify clinically relevant neurological and immunological predictors for nerve injury and reactions.

**Methodology/Principal Findings:**

The study, in two centres in India, recruited 188 new, previously untreated patients with multi-bacillary leprosy who had no recent nerve damage. These patients underwent a series of novel blood tests and nerve function testing including motor and sensory nerve conduction, warm and cold detection thresholds, vibrometry, dynamometry, monofilament sensory testing and voluntary muscle testing at diagnosis and at monthly follow up for the first year and every second month for the second year. During the 2 year follow up a total of 74 incident events were detected. Sub-clinical changes to nerve function at diagnosis and during follow-up predicted these new nerve events. Serological assays at baseline and immediately before an event were not predictive; however, change in TNF alpha before an event was a statistically significant predictor of that event.

**Conclusions/Significance:**

These findings increase our understanding of the processes of nerve damage in leprosy showing that nerve function impairment is more widespread than previously appreciated. Any nerve involvement, including sub-clinical changes, is predictive of further nerve function impairment. These new factors could be used to identify patients at high risk of developing impairment and disability.

## Author Summary

Leprosy is a disease of skin and peripheral nerves. The skin changes aid early detection and diagnosis, while the nerve damage leads to progressive impairment and disability. The aim of this study was to identify new risk factors at diagnosis and during follow-up that would predict which patients would develop nerve damage. The study, in two centres in India, recruited 188 new previously untreated patients with multi-bacillary leprosy who had no recent nerve damage. These patients underwent a series of novel blood tests and nerve function testing as diagnosis and at monthly follow up. Evidence of sub-clinical changes to nerve function at diagnosis and during follow-up predicted new nerve damage. None of the blood tests at diagnosis predicted further nerve damage while changes to one serological marker, TNF alpha, before an event were predictive. These findings increase our understanding of the processes of nerve damage in leprosy showing that nerve function impairment is more widespread than previously appreciated and that any nerve involvement is predictive of further nerve function impairment. These new factors could be used to identify patients at high risk of developing impairment and disability.

## Introduction

Leprosy is a disease of skin and peripheral nerves, as well as other organs, resulting from the interaction between *Mycobacterium leprae* and the host response. The involvement of the skin aids early detection and diagnosis while the injury to the motor, sensory and autonomic function of peripheral nerves leads to progressive impairment of both structure and function. Nerve injury occurs gradually through the course of the disease as well as acutely in association with rapid changes in immune responses commonly known as reactions. Over the past decade a number of important cohort studies in Ethiopia [1] , Thailand [2] and Bangladesh [3] have advanced our knowledge of the epidemiology of and clinical risk factors for neuropathy and reactions in leprosy. Multibacillary forms of leprosy, increasing age, and evidence of existing nerve impairment are strongly predictive of new nerve function impairment and reactions which have a peak in occurrence 3–4 months after starting chemotherapy. A clinical prediction rule based on type of leprosy and presence of nerve function impairment neatly summarised the key risk factors [4].

Similarly understanding of the mechanisms of nerve injury and reactions in leprosy has increased over the past decade starting with the potential processes by which *M.leprae* binds to laminin in the basement lamina of the Schwann cell [5],[6]. The uptake of *M.leprae* by Schwann cells is associated with an inflammatory response and local oedema leading to demyelination [7]. Further work is needed to elaborate the mechanisms of nerve injury and reactions in leprosy.

Recent trials in Nepal and Bangladesh [8] have investigated prevention of nerve damage and reactions using low dose steroids. This research demonstrated a reduction in the incidence of new reactions and nerve function impairment in the short term but this effect was not sustained at one year. The reduction was greater in those with no nerve function impairment at diagnosis. Treatment trials of reactions using steroids have shown that long courses are better than shorter courses but their effectiveness is limited [9] while other studies have demonstrated that recovery can occur spontaneously without treatment [10].

Further studies combining both basic research and epidemiology are needed to elucidate the mechanisms of nerve damage and reactions. The INFIR (ILEP Nerve Function Impairment and Reactions) Cohort Study [11] was established to identify clinically relevant neurological and immunological predictors for nerve injury and reactions both at diagnosis and immediately preceding events. New evidence on the risk factors that predict future neuropathy and reactions will contribute to understanding mechanisms as well as being clinically important in targeting follow-up of high risk individuals and in the development of strategies for early detection and prevention. The aim of this study is to assess whether novel haematological, immunological, and neurological parameters measured at diagnosis and prior to events can predict neuropathy and reaction events in patients with newly diagnosed untreated multibacillary leprosy independently of established clinical risk factors.

## Methods

Newly diagnosed, previously untreated, multibacillary leprosy patients were recruited at one of two specialist leprosy referral centres (The Leprosy Mission Hospitals at Naini and Faizabad) in Uttar Pradesh, North India. Patients who were slit skin smear positive and/or had six or more skin lesions and/or had involvement of two or more nerve trunks were eligible for inclusion. Eligible patients were invited to participate through a process of informed consent and commenced on a full course of multibacillary (MB) multidrug therapy (MDT). A standardised history using a checklist was taken from all patients recruited to the study and a clinical and neurological examination conducted which included nerve function assessment with motor and sensory nerve conduction testing, warm and cold detection thresholds, vibrometry, dynamometery, monofilament and voluntary muscle testing [11]. Blood sampling and a skin biopsy was performed at recruitment and the specimens were analysed at the Stanley Browne Laboratories in Miraj, Maharashtra and LEPRA Blue Peter Research Centre in Hyderabad. The blood samples were assayed by ELISA for anticeramide and S100 antibodies (Sigma Chemicals, USA), and for antibodies against LAM (lipoarabinomannan) and PGL-1 (phenolic glycolipid 1) provided by Professor Brennan, USA. Serum TNF-alpha was also estimated by ELISA. The patients were followed up monthly for one year and every second month during the second year when repeat nerve function assessment and blood samples were collected.

The outcome events for the analysis presented here included a first occurrence of neuritis, type 1 or reversal reaction, type 2 or erythema nodosum leprosum, new sensory impairment or new motor impairment based on clinical assessments [11]. Excluded were patients exhibiting signs of reaction at diagnosis or reporting recent onset changes in sensory or moter nerve function, recent being defined as duration of 6 months or less. The analysis of risk factors was based on two categories of outcomes – all first incident events (ALL) and first incident events which included new nerve function impairment (NFI). These categories were selected based on expected numbers of events sufficient to conduct analyses and providing categories that represented sufficiently distinct groups. Some patients experienced multiple recurrent or chronic events during follow-up. Identification of risk factors for these outcomes is beyond the scope of the present paper.

The factors included in the analysis were grouped into two types, established factors and novel factors which had not been formally tested in a large enough cohort study. The established risk factors were based on the findings of previous cohort studies [1]–[3] and which are clinically and routinely measured. In an initial analysis we assessed the predictive value of these variables for the Cohort data and identified a subset with optimal predictive value.

Novel factors are those included in this study that have not been previously assessed in terms of their ability to predict events independent of the established clinical factors. For novel neurological factors, impaired status was defined by applying reference values computed from studies of normal subjects resident in the study area but having no known neurological condition (Papers submitted). Using Cox Proportional Hazard Regression analysis, univariate hazard ratios were calculated for all novel factors at diagnosis and at the assessment immediately prior to the incident event. Hazard ratios for factors measured at two assessments before an event and the change between one and two assessments prior to an event were also calculated, though inevitably this data was unavailable for the 15 patients who had an incident event diagnosed at the first follow-up after diagnosis. Risk factors which showed a hazard ratio that was significantly different from 1 were re-analysed, adjusting for the optimal subset of established risk factors described above.

The overall sample size calculation for the Cohort Study was based on a risk factor present in 20% of the study population and an outcome frequency in the unexposed of 5%. A sample size of 240 was needed to detect a relative risk of 4 and 200 to detect a 20% difference between predictive values. A sample size of 300 was planned based on an estimated loss to follow-up of 10–20%. The exclusions from the analysis presented here caused a reduction in sample size and inevitable reduction in statistical power. Our presentation of results therefore focuses on findings that were replicated across nerves and across nerve functions.

### Ethical Considerations

Ethical approval for the study was given by the Research Ethics Committee of the Central JALMA Institute for Leprosy in Agra. Informed, written consent was obtained from the study participants before inclusion in to the study using a standard consent form. No financial incentives were given to participants however travel expenses were refunded and where relevant compensation for lost earnings for daily workers.

## Results

### The Cohort

The INFIR study cohort recruited 303 new MB leprosy patients, including 115 who had reactions or new neuropathy at the time of diagnosis. The analysis identifying risk factors for a first incident event is therefore based on the 188 patients with no baseline reactions or new neuropathy within the 6 months prior to diagnosis. 78.6% completed MDT and 12 months follow-up. Most were male (71.8%). 143 (76%) had self-reported signs and symptoms of leprosy of more than 6 months duration including 79 (42%) for more than one year. Most were classified as Borderline Tuberculoid BT (66.0%) by Ridley Jopling classification, the remainder were Borderline Lepromatous BL (23.4%) and Lepromatous Lepromatous LL (10.6%). 28% had positive slit skin smear tests and 72% negative. Most had evidence of at least one enlarged nerve (81.4%) and 28% had more than 4 enlarged nerves.

### Incident Events

**Figure 1:**
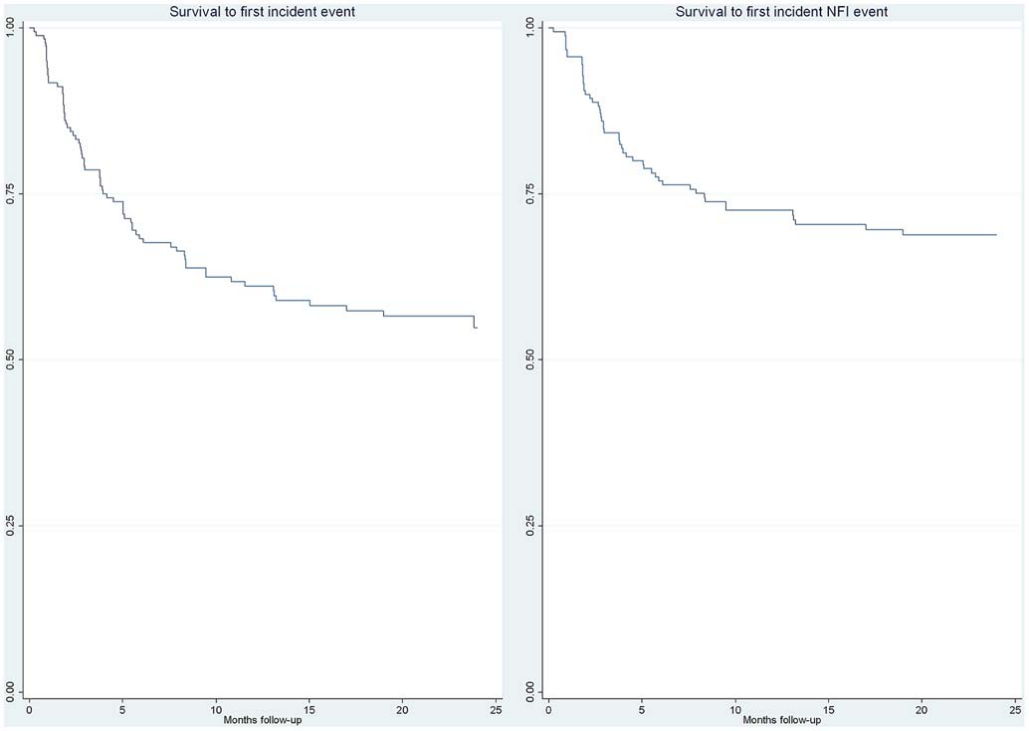
Kaplan-Meier survival to first incident event and to first event with new NFI.

74 first incident events were detected through routine assessment and self reporting based on clinical examination including clinical nerve function assessment by sensory and voluntary muscle testing. The survival curve is shown in Figure 1 where 62.2% of first events occurred in the first 4 months and only 7 first events occurred in the second year of follow-up. Of the 74 events, 69 were classed as type 1 (reversal reactions) and 5 as type 2 (ENL reactions), and in 54 the event included a change in nerve function.

### Clinical and Demographic Factors and the Risk of New Events

Seventy four (39.4%) patients (ALL) out of the cohort of 188 had a reaction or nerve function impairment event, in 54 patients this included new nerve function impairment (NFI) . The hazards ratios for the first event (ALL and NFI) for the clinically assessed factors from the history and routine examination are presented in Table 1. The risk of an event increased significantly with increasing age and those having an event were on average 6.5 years older than those who remained event free but there was no difference in risk between men and women. There was little difference in the event rate by clinical classification being 39.5% in BT, 40.9% in BL, and 35.0% in LL patients, and no relationship with being skin smear positive or delay in diagnosis was observed. Nerve enlargement was significantly associated with increased risk of events, both ALL events and NFI events. Having a higher number of skin lesions was associated with increased risk of an event but this was not statistically significant. On the basis of these observations age, sex, nerve enlargement and number of skin lesions were identified as the optimal set of covariates to include in the analyses assessing the predictive value of novel factors. Clinically measured nerve function impairment was not used as a covariate as this was the method used to diagnose new events. Haemoglobin levels and a white cell count at diagnosis were not predictive of events.

**Table 1:** Clinical risk factors, numbers at risk and univariate hazard ratios and 95% confidence intervals for ALL new events and for new nerve function impairment events (NFI).

	Numbers at risk, hazard ratios and confidence intervals		
	N	All Events	NFI Events
**Age**			
Up to 25	69	1	1
26–40	73	1.29 (0.72–2.34)	1.51 (0.69–3.33)
41+	46	2.29 (1.28–4.08)	3.90 (1.88–8.09)
**Sex**			
Male	135	1	1
Female	53	0.93 (0.56–1.53)	0.97 (0.53–1.76)
**Nerve Enlargement**			
More than 4	21	1.15 (1.01–1.31)	1.23 (1.06–1.44)
Lesions			
0–5	34	1	1
6–10	39	1.74 (0.78–3.88)	1.42 (0.59–3.42)
More than 10	115	1.54 (0.76–3.15)	1.15 (0.53–2.49)

ALL includes all new skin and nerve function impairment events of both reversal and type 2 reactions in the 188 patients with no recent events at diagnosis. NFI includes only events which had new nerve function impairment.

### Novel Risk Factors Assessed at Diagnosis

#### Neurological factors

Cox proportional hazard ratios for any new event for abnormalities in motor or sensory nerve conduction velocity, latency or amplitude, for loss warm or cold thermal sensation or reduced vibration perception for each nerve trunk tests were calculated, including adjustments for age, sex, nerve enlargement and number of skin lesions. All those reaching statistical significance are reported here.

**Figure 2:**
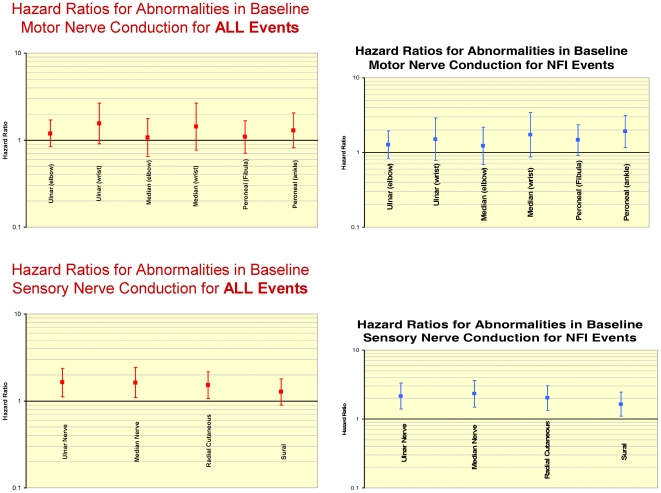
Adjusted hazard ratios attached to assessments of baseline motor and sensory nerve conduction for ALL and NFI outcome events.

Abnormalities in motor nerve conduction in the ulnar nerve (above the elbow and at the wrist), the median nerve (at the elbow and at the wrist) and the peroneal nerve (at the fibular head and at the ankle) were associated with an increased risk but none were statistically significantly predictive of any new event. Abnormalities in sensory nerve conduction in the ulnar, median and radial cutaneous nerves but not the sural nerve were significantly associated with new events (Figure 2). Only ulnar sensory nerve conduction and radial cutaneous sensory nerve conduction were statistically significant after adjustment. Abnormality in warm sensation in the skin areas supplied by the ulnar nerve and the posterior tibial nerve were significant predictors of new events but were not significant after adjustment. Abnormality in vibration sensation in the ulnar, median, radial cutaneous, posterior tibial and sural nerves were not predictive of future events.

The same Cox proportional hazard analyses were conducted for the same neurological variables this time predicting nerve function impairment events (Figure 2). Abnormalities in motor nerve conduction tended to be associated with an increased risk of an NFI event but this was only significant for the peroneal nerve at the ankle and this was not significant after adjustment. Abnormalities in sensory nerve conduction in all nerves tested were significantly associated with an increased risk of a new nerve function impairment with hazard ratios of around 2, and all remained significant after adjustment except median nerve conduction amplitude and latency in the sural nerve. Abnormalities of warm sensation in the ulnar, radial cutaneous, posterior tibial and sural nerves were predictive for new NFI events, but only in the posterior tibial nerve was this significant after adjustment. Abnormalities in cold sensory function in the radial cutaneous, posterior tibial and sural nerves were predictive for new NFI events but only in the sural nerve did this remain significant after adjustment. Only abnormality in vibration sensation in the ulnar nerve was predictive of new NFI events but this was not significant after adjustment.

#### Immunological and serological

The hazards ratios for both ALL new events and for nerve function impairment (NFI) events for serum anticeramide, serum TNF alpha, PGL 1 IgG and IgM, serum S100, and serum LAM IgG are presented in Table 2. None are significantly different from 1.

**Table 2:** Hazard ratios for abnormalities in serology and immunology for ALL events and for NFI events.

	Hazard Ratios and 95% confidence intervals for ALL and NFI outcomes attached to impaired values for serological markers at baseline.		
	N, median, IQR	ALL events	NFI Events
**Anticeramide**	186, 0.62, 0.36–0.99	1.17 (0.67–2.04)	1.02 (0.51–2.03)
**S100**	188, 53.0, 34.5–85.0	0.80 (0.49–1.30)	0.65 (0.35–1.20)
**TNF Alpha**	188, 2.14, 0.71–17.19	0.73 (0.36–1.46)	0.69 (0.29–1.61)
**PGL1_IgG**	188, 63.5, 29.50–106.95	1.34 (0.81–2.20)	1.07 (0.60–1.90)
**PGL1_IgM**	188, 66.5, 38–120	1.29 (0.79–2.10)	1.01 (0.57–1.77)
**LAM_IgG1**	188, 1.0, 0–15	0.90 (0.56–1.46)	1.02 (0.58–1.79)
**LAM_IgG3**	188, 4.0, 3–10	0.96 (0.60–1.54)	0.99 (0.57–1.74)

### Novel Risk Factors Assessed Immediately before a New Event

#### Neurological

**Figure 3:**
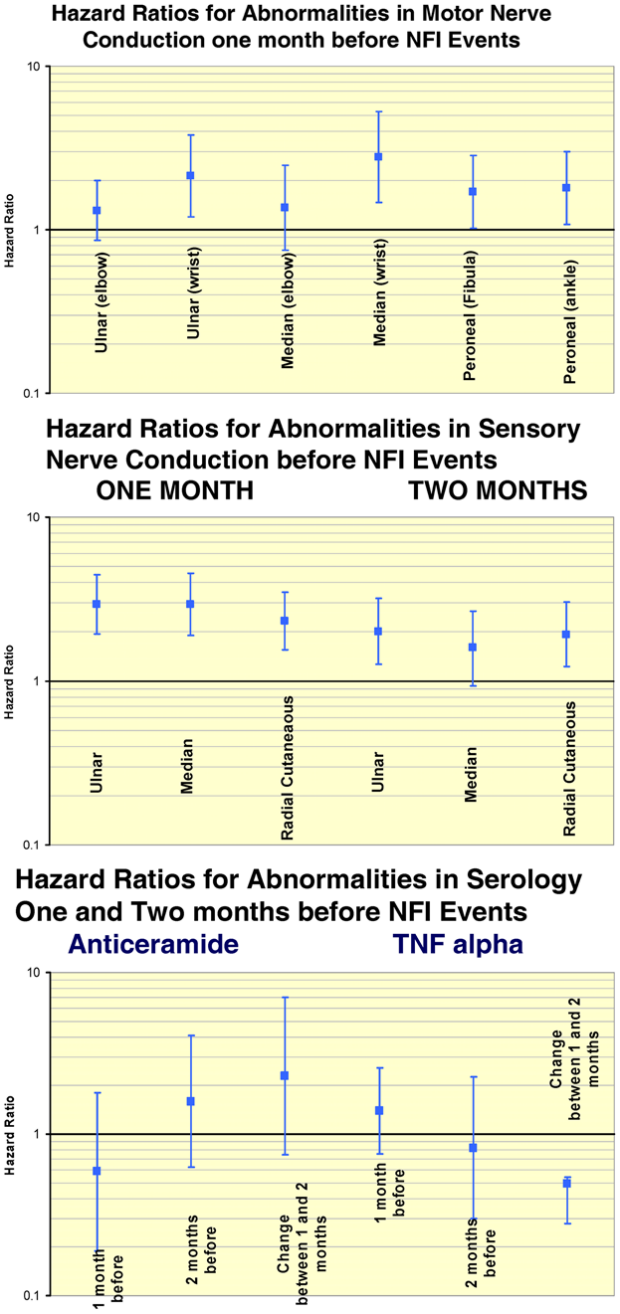
Adjusted hazard ratios for new nerve function impairment events for motor and sensory conduction, and serology abnormalities before an event.

Abnormalities in motor nerve conduction in the assessment prior to a new NFI event showed a trend across the nerves tested to predict the event and the effect of abnormalities in sensory nerve conduction was similar but stronger than that for motor nerve conduction. In sensory nerve conduction the hazard ratios for an NFI event were higher in the assessment immediate preceding the event compared to the one before as seen in Figure 3 for the median, ulnar, and radial cutaneous nerves.. Hazard ratios for abnormalities in motor and sensory nerve conduction in the assessment before an event were greater for NFI events than for ALL events.

#### Immunological and serological

The serological assays prior to an event were not predictive of the event however change in TNF alpha between the assays one and two assessments before an event was statistically significantly (p = 0.009) different from one (Figure 3).

## Discussion

This is an important study which comprehensively assesses novel risk factors for nerve function impairment and reactions in leprosy using rigorous methods and extensive and careful follow-up over 24 months. The study builds on existing knowledge of clinical and demographic risk factors for reactions and for new nerve function impairment occurring during and after MDT. Using data from the INFIR Cohort Study, the analysis focuses on the 188 patients with newly diagnosed multibacillary leprosy who had no recent nerve function impairment or reactions at the time of diagnosis. The clinical and demographic risk factors demonstrated a level of risk similar to other cohort studies [1]–[3] recognising that this cohort was restricted to multibacillary patients so that bacteriological index, Ridley-Jopling classification and number of lesions were less predictive in this more homogenous cohort. None of the haematological factors measured were predictive of events.

### Predictive value of neurological status at diagnosis

Abnormalities in motor nerve conduction at diagnosis showed a tendency towards increased risk for ALL events. While individually these failed to reach statistical significance, a similar pattern was found for both latency and amplitude in all nerves tested. There was a stronger effect for ALL events seen with sensory nerve conduction abnormalities at diagnosis compared to motor nerve conduction with ulnar and radial cutaneous nerve conduction remaining statistically significant after adjustment, the pattern for amplitude and latency was similar. Abnormalities in warm and cold thresholds at diagnosis were predictive of ALL events but the effect disappeared after adjustment, any effect of vibration sensation also disappeared on adjustment.

When the analysis was restricted to the 54 patients whose new event included nerve function impairment both motor and sensory nerve conduction were more strongly predictive, particularly sensory nerve conduction where hazard ratios were around 2 and remained significant after adjustment. Any effect of abnormality in thermal and vibration sensation disappeared after adjustment. These motor and sensory nerve conduction findings confirm that any nerve function impairment at diagnosis is predictive of events, particularly events which include new nerve function impairment. This confirms the clinico-pathological observation that inflammation in leprosy once established is very difficult to switch off [12]. However the widespread nature of the effect is important as the effect is seen across all peripheral nerve tested. The apparent predictive effect of vibration and thermal sensation disappears on adjustment largely due to age as a confounding factor as has been observed elsewhere [13].

### Predictive value of serological status at diagnosis

The serological measures selected for this study were those that had been associated with bacterial load [14], nerve damage [15] and reactions [16] in leprosy in previous studies. Measures of LAM antibody and anti PGL1 at diagnosis were inter-correlated and related to bacteriological index but they did not predict events when measured at diagnosis in this multibacillary cohort. Measures of S 100 and anticeramide were also inter-correlated (r = 0.40) but they were not related to the extent of nerve damage or to reactions at diagnosis and did not predict future events.

### Predictive value of neurological status prior to an incident event

The pattern of effect of abnormalities in motor and sensory nerve conduction immediately prior to an event was similar to that at diagnosis where sensory nerve conduction had a stronger and more consistent effect than motor nerve conduction, and the effect was stronger for NFI events than for ALL events. Abnormalities in sensory nerve conduction one month prior to an event have a stronger effect than two months prior to the event. An analysis of early diagnosis of neuropathy in leprosy in this study cohort reported that sensory nerve conduction was the most frequent and earliest affected test [17]. These findings may be influenced by the staging of symptoms that varies between individual nerves and the variation in reported duration of symptoms prior to diagnosis.

### Predictive value of serological status prior to an incident event

A change in TNF alpha levels rather than the absolute level prior to an event was predictive, consistent with the findings in previous smaller studies [18],[19]. The levels of expression of TNF alpha showed considerable individual variation and the rate of change appeared to be the important predictive factor rather than the absolute level.

This carefully conducted cohort study has confirmed the importance of the clinical risk factors of age, classification, and pre-existing nerve damage as predictors of new nerve function impairment and reactions. Abnormalities in motor and sensory nerve conduction at diagnosis and before events have been demonstrated to be predictive of future events. Sensory nerve conduction is a stronger predictor than motor nerve conduction and is independent of the established clinical risk factors, particularly events that include nerve function impairment. Thermal and vibration sensation shows some evidence of being predictive but this appears to be confounded by age. Serological measures of antibodies to nerve components and to *M.leprae* cell wall antigens are not predictive of new events but changes in TNF alpha levels occur prior to new events. These findings expand our understanding of the process of nerve function impairment in leprosy showing that nerve involvement is much more widespread and occur earlier than previously understood, and that any nerve involvement is predictive of further impairment. These parameters can be used to identify individual patients at high risk of developing further nerve damage.

## Supporting Information

Checklist S1 STROBE checklist.
(0.06 MB DOC)
